# Comparison of the Diagnostic Utility of Manual Screening and the ThinPrep Imaging System in Liquid-Based Cervical Cytology*

**DOI:** 10.5146/tjpath.2019.01473

**Published:** 2020-05-15

**Authors:** Zühal Özcan, Elife Kımıloğlu, Ayşenur Akyıldız İğdem, Nusret Erdoğan

**Affiliations:** Department of Pathology, Haydarpasa Numune Education and Research Hospital, Istanbul, Turkey; Gaziosmanpasa Taksim Education and Research Hospital, Istanbul, Turkey; Istinye University, Faculty of Medicine, Istanbul, Turkey

**Keywords:** ThinPrep Imaging System, Liquid-based cervical cytology, Manual method

## Abstract

***Objective:*** To compare the diagnostic results of the ThinPrep manual method (TPMM) and ThinPrep automated method (TPAM) in liquid-based cytology and present the advantages and disadvantages of both methods.

***Material and Method:*** A total of 1.500 randomized ThinPrep Pap tests that were screened manually and archived in 2015 were reviewed by a blinded researcher manually and by the ThinPrep automatic method.

***Results:*** There was a 83.3% increase in the detection of ASCUS (Atypical squamous cells of undetermined significance) with the TPAM compared to the TPMM, and with respect to the reference results, the accuracy was higher for the TPAM than for the TPMM. We also noted a 33.3% increase in the rate of LSIL (Low grade squamous intraepithelial lesion) and 20% increase in the rate of HSIL (High grade squamous intraepithelial lesion) by the TPAM. Concordance was best between the TPAM and reference cytologic diagnoses. The sensitivity was higher for the TPAM and the specificity was similar for both methods. The false positive rate was higher for the TPAM than the TPMM but the false negative rate was higher for the TPMM. We determined a 30% gain in screening time per smear by the TPAM. However, rejection of many samples by the system, especially because of air bubbles, was a limitation of the TPAM.

***Conclusion:*** The TPAM has advantages over the TPMM as well as disadvantages such as limiting features and a high false positive rate. The TPAM should be supported by the manual method to decrease the false positive rate.

## INTRODUCTION

Cervical cancer remains the most important public health problem in developing countries and in Turkey. It was the fourth most frequent type of cancer seen among women worldwide in 2012, and more than 500,000 new cases and 266,000 estimated deaths related to the disease have been reported every year (1). Cervical cancer is a preventable type of cancer because of the length of its pathogenetic process and the presence of a preinvasive period. Prevention and treatment of cervical cancer depend on the detection of its risk factors and the eradication of preventable ones, the realization of optimal screening tests appropriate for the age group, and the establishment of early diagnosis and treatment (2).

The Food and Drug Administration has approved many liquid-based cytology techniques in recent decades (3). These methods differ from classical Papanicolaou smears in many aspects. ThinPrep (HOLOGIC) is one such method, and the ThinPrep Imaging System (TIS) is an automated imaging and review system that selects “22 fields of view” from the samples presented to it. The manufacturers of the TIS particularly note that the system aims to help cytopathology specialists by highlighting areas of a slide for further manual review and that the product is not intended as a replacement for manual review (4). When compared with conventional cytology, the relative true positive and false positive rates are 1.13 and 1.12, respectively. This indicates that the ThinPrep test has a high sensitivity but low specificity (5). This technique is more expensive than the classical Pap smear test; however, since it is a more sensitive test, the requirement of longer between-screening intervals balances its higher cost. Liquid-based cytology can decrease the rate of false negative results arising from errors made during screening tests and from interpretation of cytological test results (6).

In the present study, we aimed to compare 1500 randomized ThinPrep slides by using the TPMM and the TPAM. Our study differs from other similar studies by using the same ThinPrep Pap smear samples to compare the two methods and the same samples for reference screening.

## MATERIALS and METHODS

The study was conducted in accordance with the principles of the Helsinki Declaration and approved by the local Institutional Review Board (08.07.2015).

### Study Design

A total of 1500 ThinPrep slides that were randomly selected and were then screened and diagnosed manually by 7 different researchers and archived in 2015 at the ‘Gaziosmanpasa Taksim Education and Research Hospital’ were included in the study. The Pap tests were rescreened manually (TPMM) then pre-screened on a ThinPrep 2000 device (Cytyc Corporation, Boxborough, MA, USA) and rescreened again only for “22 fields of view” on a TIS microscope at x10 magnification (TPAM) by the same blinded, inexperienced researcher (assistant doctor) to compare the diagnostic results and the positive/negative sides of the two methods.

### ThinPrep Imaging System

A barcode that includes the protocol number and the year data was placed on each ThinPrep slide and prescreened by the same inexperienced researcher from the above section with the TIS. Eventually, data from 22 fields of view for each slide were obtained by and stored on the system. During that process, the system refused to screen some of the slides for a variety of reasons, such as ‘bubble artifacts’. Approximately 300 pap smears that were not recognized or refused to be read by the device were kept in a xylene solution to remove the probable artifacts following careful replacement of their cover slips. Subsequently, approximately 70 pap smears were refused by the device again and therefore excluded from the study. Another 70 new pap smears were randomly selected from the database that was archived in 2015 and included in the study (this preserved the original number of slides as n=1500).

### ThinPrep Manual Method

Pap smears were rescreened on a TIS microscope by the TPMM by the same inexperienced researcher. Each day, an average of 100 slides were reviewed. Screening was performed without knowledge of the individual histopathologic results. While screening, cases were grouped into reproductive, premenopausal or perimenopausal and menopausal categories. Slides were classified according to the Bethesda 2014 classification.

### ThinPrep Automated Method

Pap smears were rescreened on a TIS microscope at x10 magnification over “22 fields of view” by the same inexperienced researcher. Each day, an average of 130 slides were reviewed. Screening was performed without knowledge of the first pathologic results or the results obtained by the TPMM. Slides were classified according to the Bethesda 2014 classification.

### Reference Diagnosis

A total of 91 cases that were diagnosed as “Epithelial cell anomaly” on the original cytopathology reports from 2015 or by the TPMM or the TPAM were screened again by an experienced consultant doctor (reference), and the new diagnoses were accepted as the gold standard. For the cases that were diagnosed with “No intraepithelial lesion or malignancy (NILM)” on the original cytopathology reports from 2015 or by the TPMM or the TPAM, the original cytopathology diagnoses from 2015 were accepted as the gold standard.

### Statistical Analysis

Data were reported as percent values as appropriate. Group comparisons were performed using Fleiss’ kappa and Krippendorff’s alpha tests. If the same results were obtained, Cohen’s kappa was calculated to determine the concordance between two tests. A two-sided p-value <0.05 was considered statistically significant. The reference values for the concordance between all three tests were as follows: Unimportant (0.20), Low (=0.20 - 0.40), Median (=0.40 - 0.60), Important (=0.60 - 0.80), and Very important (=0.80 - 1.00). For optimal statistical dual and triple concordance analyses between the results of the screening methods and the references, we excluded two cases that were diagnosed as “Atypical glandular cell (AGC)” only by the TPMM and three cases that were diagnosed as “Atypical squamous cells, cannot exclude HSIL (ASC-H)” only by the TPAM. Additionally, four cases that were diagnosed as “unsatisfactory for evaluation” by the two methods were not included in any comparison analysis.

## RESULTS

The mean age of patients corresponding to the 1500 cases reviewed was 40.0±11.47 (range, 18 to 83) years. A total of 1328 (88.5%) cases were in the reproductive period, 102 (6.8%) were premenopausal or perimenopausal, and 70 (4.7%) were menopausal. The “unsatisfactory for evaluation” rate was 0.3% for both methods (n=4).

The breakdown diagnoses was 1455 (97%) NILM, 14 (0.9%) LSIL, 7 (0.5%) HSIL, 18 (1.2%) ASCUS and 2 (0.1%) ASC-H by the TPMM; 1432 (95.5%) NILM, 19 (1.2%) LSIL, 9 (0.6%) HSIL, 33 (2.2%) ASCUS and 3 (0.2%) ASC-H by the TPAM; and 1440 (96%) NILM; 31 (2.1%) LSIL, 6 (0.5%) HSIL and 19 (1.3%) ASCUS by the reference method.

The ASC/SIL ratio was < 2 for both methods (TPMM=0.9 *vs.* TPAM=1.2).

**Figure 1:**
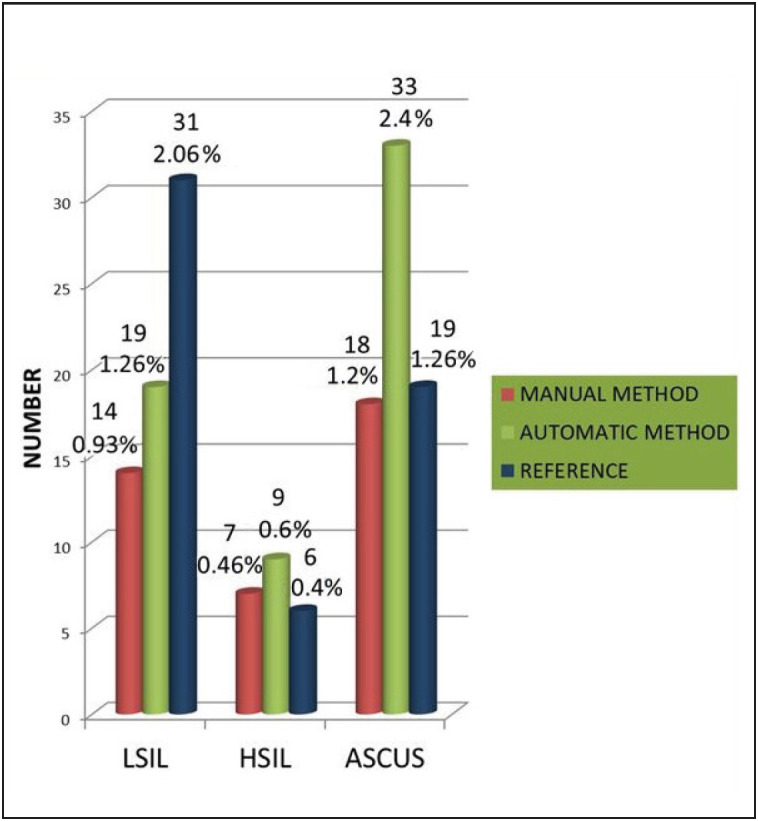
Distribution of the diagnoses of LSIL, HSIL and ASCUS with respect to the methods and reference.

Diagnostic analysis showed that 26 cases diagnosed with ASCUS, 6 with LSIL and 1 with HSIL by the TPAM were all diagnosed with NILM by the TPMM (Table 1 Table 2). There was no distinct difference between the LSIL diagnoses for the two methods. However, 7 cases were diagnosed with NILM, 3 with LSIL and 1 with HSIL by the TPAM were all diagnosed with ASCUS by the TPMM. There was an 83.3% increase in the detection of ASCUS (n=33 *vs.* 18) and a 33.3% increase in the detection of LSIL (n=19 *vs.* 14) and a 20% increase in the detection of HSIL (n=9 *vs. *7) by the TPAM versus TPMM (Figure 1). With respect to the reference results, the accuracy was higher for the TPAM than the TPMM (Table 3, Table 4, Table 5, Table 6).

**Table 1:** Diagnostic comparison between the TPMM and the TPAM.

			**TPMM Smear Diagnosis**				**Total**
			**NILM**	**LSIL**	**HSIL**	**ASCUS**	
**TPAM** **Smear Diagnosis**	NILM	n	1422	1	0	7	1430
		%	95.3	0.1	0.0	0.5	96
	LSIL	n	6	10	0	3	19
		%	0.4	0.6	0.0	0.2	1.2
	HSIL	n	1	1	6	1	9
		%	0.1	0.1	0.4	0.1	0.6
	ASCUS	n	26	1	1	5	33
		%	1.7	0.1	0.1	0.3	2.2
**Total**		n	1455	13	7	16	1491
		%	97.5	0.9	0.5	1.1	100.0

*Crosstab. Cohen’s Kappa Test.* **NILM:** Negative for intraepithelial lesion or malignancy; **ASCUS:** Atypical squamous cells of undetermined significance; **LSIL:** Low-grade squamous intraepithelial lesion; **HSIL:** High-grade squamous intraepithelial lesion; **TPAM:** ThinPrep automated method; **TPMM:** ThinPrep manual method.

**Table 2:** Symmetric measures of the cross-tabulation analysis for Table 1.

		**Value**	**Asymp. Std. Error^a^**	**Approx. T^b^**	**Approx. Sig.**
Measure of Agreement	Kappa	-.119	.094	-1.229	.219
Valid Cases (n)		78			

a. Not assuming the null hypothesis. b. Using the asymptotic standard error assuming the null hypothesis.

**Table 3:** Diagnostic comparison between the TPMM and reference.

			**Reference Smear Diagnosis**				**Total**
			**NILM**	**LSIL**	**HSIL**	**ASCUS**	
**TPMM** **Smear Diagnosis**	NILM	n	1434	5	1	15	1455
		%	95.9	0.3	0.1	1	97.3
	LSIL	n	0	13	0	1	14
		%	0.0	0.9	0.0	0.1	1
	HSIL	n	0	3	4	0	7
		%	0.0	0.2	0.3	0.0	0.5
	ASCUS	n	5	10	0	3	18
		%	0.3	0.7	0.0	0.2	1.2
**Total**		n	1439	31	5	19	1494
		%	96.4	2	0.3	1.3	100.0

*Crosstab. Cohen’s Kappa Test.* **NILM:** Negative for intraepithelial lesion or malignancy; **ASCUS:** Atypical squamous cells of undetermined significance; **LSIL:** Low-grade squamous intraepithelial lesion; **HSIL:** High-grade squamous intraepithelial lesion; **TPMM:** ThinPrep manual method.

**Table 4:** Symmetric measures of the cross-tabulation analysis for Table 3.

		**Value**	**Asymp. Std. Error^a^**	**Approx. T^b^**	**Approx. Sig.**
Measure of Agreement	Kappa	.300	.102	2.862	.004
Valid Cases (n)		78			

a. Not assuming the null hypothesis. b. Using the asymptotic standard error assuming the null hypothesis.

**Table 5:** Diagnostic comparison between the TPAM and reference.

			**Reference Smear Diagnosis**				**Total**
			**NILM**	**LSIL**	**HSIL**	**ASCUS**	
**TPAM** **Smear Diagnosis**	NILM	n	1421	3	2	6	1432
		%	95.3	0.2	0.1	0.4	96
	LSIL	n	2	16	0	1	19
		%	0.1	1.1	0.0	0.1	1.2
	HSIL	n	0	6	3	0	9
		%	0.0	0.4	0.2	0.0	0.6
	ASCUS	n	17	3	1	12	33
		%	1.1	0.2	0.1	0.8	2.2
**Total**		n	1440	28	6	19	1493
		%	96.5	0.9	0.4	1.1	100.0

*Crosstab. Cohen’s Kappa Test.* **NILM:** Negative for intraepithelial lesion or malignancy; **ASCUS:** Atypical squamous cells of undetermined significance; **LSIL:** Low-grade squamous intraepithelial lesion; **HSIL:** High-grade squamous intraepithelial lesion; **TPAM:** ThinPrep automated method.

**Table 6:** Symmetric measures of the cross-tabulation analysis for Table 5.

		**Value**	**Asymp. Std. Error^a^**	**Approx. T^b^**	**Approx. Sig.**
Measure of Agreement	Kappa	-.022	.109	-.195	.846
Valid Cases (n)		78			

a. Not assuming the null hypothesis. b. Using the asymptotic standard error assuming the null hypothesis.

Concordance was worst between the TPAM and TPMM diagnoses (p<0.05 and kappa value=0.495) and best between the TPAM and reference cytologic diagnoses (p<0.05 and kappa value=0.631) (Table 7 Table 8 Table 9).

**Table 7:** Diagnostic concordance between the TPAM and the TPMM.

**Kappa**	**Standard deviation**	***p***
0.495	0.044	0.000

**TPAM:** ThinPrep automated method; **TPMM:** ThinPrep manual method; p-value <0.05; Unimportant (0.20), Low (=0.20 - 0.40), Median (=0.40 - 0.60), Important (=0.60 - 0.80), Very important (=0.80 - 1.00).

**Table 8:** Diagnostic concordance between the TPMM and reference.

**Kappa**	**Standard deviation**	***p***
0.565	0.052	0.000

**TPMM:** ThinPrep manual method; p-value <0.05; Unimportant (0.20), Low (=0.20 - 0.40), Median (=0.40 - 0.60), Important (=0.60 - 0.80), Very important (=0.80 - 1.00).

**Table 9:** Diagnostic concordance between the TPAM and reference.

**Kappa**	**Standard deviation**	***p***
0.631	0.048	0.000

**TPAM:** ThinPrep automated method; p-value <0.05; Unimportant (0.20), Low (=0.20 - 0.40), Median (=0.40 - 0.60), Important (=0.60 - 0.80), Very important (=0.80 - 1.00).

The reference diagnoses accepted as the gold standard were used to calculate sensitivity, specificity, and the false positive and false negative rates. The sensitivity was higher for the TPAM (for TPMM=62.5% *vs.* for TPAM=80%), and the specificity was similar for both methods (for TPMM=99.6% *vs.* for TPAM=98.7%). The false positive rate was higher for the TPAM than the TPMM (for TPMM=0.4% *vs.* for TPAM=1.3%), but the false negative rate was higher for the TPMM (for TPMM=37.5% *vs.* for TPAM=20%) (Table 10 Table 11).

**Table 10:** Rate-calculating template for the TPMM.

			**Reference Smear Diagnosis**		**Total**
			**+**	**-**	
**TPMM** **Smear Diagnosis**	**+**	n	35	6	41
	**–**	n	21	1.434	1.455
**Total**		n	56	1.440	1.496

‘+’ *for ASCUS, LSIL, HSIL, ASC-H, AGC and* ‘-’ *for NILM* (**NILM:** Negative for intraepithelial lesion or malignancy; **ASCUS:** Atypical squamous cells of undetermined significance; **LSIL:** Low-grade squamous intraepithelial lesion; **HSIL:** High-grade squamous intraepithelial lesion; **TPMM:** ThinPrep manual method).

**Table 11:** Rate-calculating template for the TPAM.

			**Reference Smear Diagnosis**		**Total**
			**+**	**-**	
**TPAM** **Smear Diagnosis**	**+**	n	45	19	64
	** –**	n	11	1.431	1.432
**Total**		n	56	1.440	1.496

‘+’ *for ASCUS, LSIL, HSIL, ASC-H, AGC and* ‘-’ *for NILM* (**NILM:** Negative for intraepithelial lesion or malignancy; **ASCUS:** Atypical squamous cells of undetermined significance; **LSIL:** Low-grade squamous intraepithelial lesion; **HSIL:** High-grade squamous intraepithelial lesion; **TPAM:** ThinPrep automated method).

We determined a 30% decrease in screening time per smear by the TPAM. However, the rejection of many samples by the system, especially because of air bubbles, is a limitation of the TPAM.

## DISCUSSION

In this study, we attempted to compare the manual and automated ThinPrep methods. Our study showed that the TPAM has advantages and disadvantages compared to the TPMM, such as limiting features and a high false positive rate, and we conclude that the TPAM should be supported by the manual method to decrease the false positive rate.

It has been shown that the number of atypical smear diagnoses increase when using TIS (5,6). In studies that compared the TPMM and the TPAM, similar results for ASCUS, LSIL and HSIL diagnoses were noted (7–9). In the present study, we noted a 33.3% increase in the rate of LSIL and a 20% increase in HSIL by the TPAM compared to the TPMM, but with respect to the reference results, the false positive rate was higher for the TPAM than the TPMM. As a result, the TPMM seems to be more valuable than the TPAM in this respect. Additionally, there was an 83.3% increase in the detection of ASCUS with the TPAM compared to the TPMM; however, with respect to the reference results, accuracy was higher for the TPAM than the TPMM. The concordance was best between the TPAM and reference cytologic diagnoses. The low concordance between the TPMM and reference diagnoses may be related to the evaluations being performed by an inexperienced and an experienced pathologist, respectively. In the present study, in two cases diagnosed as ‘ASCUS’ by the TPAM, candida was detected by TPMM. Therefore, screening only with “22 fields of view” can be a reason for the increased false positive rate for TPAM. The ASC/SIL ratio is a quality control method for gynecologic cytology results and is typically between 2-3. The CAP (College of American Pathologists) identifies the median of the ASC/SIL ratio as 1.7. In our study, the ratio was < 2 for both methods. Similar studies have reported a range of values between 0.74 and 2.25 (7,8,10,11). Satisfactoriness is the only quality control method of the Bethesda System. The “unsatisfactory for evaluation” rate was 0.3% for both methods in our study. In the literature, varying results of this rate have been noted (between 0.3% and 3%) (7,8,12–16). Studies in the literature mainly consist of a great number of cases but used Pap smears screened by different cytotechnologists at different times and which were rescreened by different cytotechnologists to compare the different methods (7,8,17). Our study differentiates from the others by using the same ThinPrep Pap smear samples to compare the two methods and to perform reference screening.

Renshaw and Elsheikh investigated the correlation between the sensitivity for HSIL in the TIS and the epithelial cell abnormality (ECA)-adjusted workload and showed that the performance of the TIS at the threshold for HSIL and above was negatively correlated with the ECA-adjusted workload (9). Kitchener et al. argued that monotony could have been a contributing factor in reduced vigilance while screening by the TPAM (13). Lozano et al. mentioned that automated screening causes cytotechnology operators to be more fastidious in their analyses (8). In the present study, we reviewed 12 smears per hour with the TPMM and 16 smears per hour with the TPAM.

The most significant methods of clinical efficiency in screening are specificity and sensitivity. In the literature, some of the studies accepted follow-up biopsies or TPMM results performed in different years as a reference and reported differing results regarding sensitivity and specificity (12,13,18). In the present study, sensitivity was higher for the TPAM than the TPMM, and specificity was similar for both methods. Despite of screening with only by “22 fields of view” with the TPAM, the sensitivity and specificity values were high for the TPMM. Additionally, the high false positive rate for the TPMM compared to the TPAM and the similar results for accuracy and negative predictive value between the two tests favor the use of the TPAM.

Colakkadioglu and Erkilic stated that smear slides were rejected while screening with the automated system because of the presence of blood, a small number of cells and air bubbles (19). In the present study, we excluded a total of 70 slides from the study mainly because of the presence of air bubbles. It must be emphasized that smear rejection by the device is an important limitation of TIS.

We are aware that there are clear limitations in the case series presented here. The main limitation of our study was its retrospective design. The second limitation is that the results were restricted to the outcomes from a single institution. Third, some details of the patients’ history and factors that may influence the outcome may not have been completely documented. Due to these restrictions, the associations presented here should be interpreted with caution.

In conclusion, the TPAM has advantages over the TPMM as well as disadvantages such as limiting features and a high false positive rate. The TPAM should be supported by the manual method to decrease the false positive rate.

## Conflict of Interest

The authors declare no conflict of interest.
